# Association between the Dietary Index for Gut Microbiota and periodontitis: mediation by systemic inflammation

**DOI:** 10.3389/fnut.2025.1612199

**Published:** 2025-08-13

**Authors:** J. H. Zhu, Gehong Sun, Jia Guo, Yongjuan Li, Yichao Jing, Zhen Zhang, Wenting Pan

**Affiliations:** ^1^The First Affiliated Hospital of Zhengzhou University, Zhengzhou, Henan, China; ^2^Department of Stomatology, The First Affiliated Hospital of Zhengzhou University, Zhengzhou, Henan, China

**Keywords:** Dietary Index for Gut Microbiota, periodontitis, systemic inflammation, NHANES, mediation analysis

## Abstract

**Objective:**

This study aimed to explore the association between the Dietary Index for Gut Microbiota (DI-GM) and periodontitis, and to investigate the mediating role of systemic inflammation in this relationship.

**Methods:**

We performed a cross-sectional analysis using data from the National Health and Nutrition Examination Survey (NHANES) 2009–2014, including 9,022 participants. DI-GM scores were derived from 14 dietary components known to influence gut microbiota. Periodontitis was defined using a reduced version of the Centers for Disease Control and Prevention and American Academy of Periodontology criteria. Multivariable logistic regression and restricted cubic spline (RCS) analyses were employed to assess the association between DI-GM and periodontitis. Additionally, mediation analysis was performed to examine the contribution of systemic inflammation biomarkers to the observed associations.

**Results:**

Higher DI-GM scores were inversely associated with periodontitis prevalence, with each 1-point increase in DI-GM reducing the odds of periodontitis by 5% (95% CI: 0.92–0.97; *p* < 0.001). Participants in the highest DI-GM group had 19 and 26% lower odds of moderate and severe periodontitis, respectively, compared with the lowest group. Mediation analysis suggested modest mediation effects for systemic inflammation biomarkers, particularly CRP (8.1%) and WBC (5.5%), indicating that systemic inflammation may partially explain the observed associations.

**Conclusion:**

Our findings indicate that greater adherence to DI-GM is associated with lower periodontitis prevalence, partly mediated by systemic inflammation, highlighting dietary modulation of gut microbiota as a potential strategy for periodontal disease prevention.

## 1 Introduction

Periodontitis is a chronic inflammatory disease characterized by progressive destruction of the tooth-supporting tissues, which ultimately leads to tooth loss and contributes to a variety of systemic health complications (1). According to the Global Burden of Disease Study 2021, severe periodontitis affects approximately 1.07 billion people globally, with an age-standardized prevalence rate of 12,500 cases per 100,000 population, making it the sixth most prevalent chronic disease worldwide (2, 3). Beyond its oral health consequences, periodontitis has been epidemiologically linked to several systemic conditions, including cardiovascular diseases (4, 5), diabetes (6), and rheumatoid arthritis (7), imposing significant healthcare costs and negatively impacting quality of life (8, 9). While microbial dysbiosis and poor oral hygiene are well-established risk factors (10), emerging evidence suggests that modifiable lifestyle factors (11), particularly dietary habits (12, 13), may also influence periodontitis development and progression.

The Dietary Index for Gut Microbiota (DI-GM) is a comprehensive dietary assessment tool developed from an extensive review of the literature (14), designed to quantify the impact of dietary patterns on gut microbiota composition and diversity. Diets with high DI-GM scores emphasize fiber-rich, polyphenol-containing, and fermented foods known to promote microbial diversity and short-chain fatty acid (SCFA) production—compounds that exhibit anti-inflammatory and immunoregulatory effects (15, 16). Conversely, low DI-GM scores, which reflect high intake of saturated fats and refined carbohydrates, promote systemic inflammation and microbial dysbiosis, thereby creating a pro-inflammatory milieu that accelerates periodontal tissue degradation (17, 18). Although previous studies have linked DI-GM with reduced risks of diabetes (19), stroke (20), and other disease (21), its relationship with periodontitis remains unexplored.

Systemic inflammation, commonly reflected by biomarkers such as C-reactive protein (CRP) and white blood cell count (WBC), has been implicated in both the onset and progression of periodontitis (22). The gut-oral axis refers to the bidirectional interplay between gut microbiota and oral immune responses, mediated through systemic metabolites and inflammatory pathways (23). Through this axis, dietary modulation of gut microbiota may influence systemic inflammation, whereby microbial metabolites such as SCFAs and endotoxins regulate host immune activity at distant sites, including the oral cavity (24, 25). Although gut microbiota composition was not directly assessed in this study, the inclusion of validated inflammatory biomarkers enables an indirect but biologically plausible examination of this pathway.

Therefore, leveraging data from the National Health and Nutrition Examination Survey (NHANES) 2009–2014, this study aims to examine the association between DI-GM and periodontitis and to investigate the mechanistic role of systemic inflammation in this relationship through mediation analysis. By elucidating these pathways, our findings may provide new insights into the role of diet in the prevention of periodontitis and its associated systemic impacts.

## 2 Method

### 2.1 Study population

This cross-sectional study was conducted using data from three consecutive survey cycles (2009–2014) of the National Health and Nutrition Examination Survey (NHANES), a nationally representative health and nutrition survey managed by the National Center for Health Statistics (NCHS) designed to evaluate the health status of the non-institutionalized U. S. population. All NHANES protocols received approval from the NCHS Research Ethics Review Board, and all participants provided written informed consent prior to inclusion.

Initially, a total of 30,468 participants from NHANES 2009–2014 were considered for inclusion. Sequential exclusion criteria were applied: age younger than 30 years, incomplete periodontal assessments or edentulism, missing dietary data across the required two-day dietary recall, and pregnancy. After these exclusions, the final analytical sample comprised 9,022 adult participants for the primary analysis. Additionally, mediation analyses involving systemic inflammation biomarkers, including C-reactive protein (CRP) and leukocyte counts, utilized subsamples ranging from 3,106 to 8,773 participants, depending on biomarker data availability. Appropriate NHANES survey weights were incorporated into all analyses to ensure nationally representative estimates.

### 2.2 Assessment of the Dietary Index for Gut Microbiota (DI-GM)

The DI-GM was specifically developed utilizing dietary data from NHANES 2009–2014, encompassing 14 dietary components: 10 microbiota-beneficial (fermented dairy, chickpeas, soybean, whole grains, fiber, cranberries, avocados, broccoli, coffee, green tea) and 4 detrimental (red meat, processed meat, refined grains, high-fat diets [≥40% total energy]) (14). For each beneficial dietary component, participants received a score of 1 if their intake exceeded the sex-specific median, otherwise 0. Conversely, for detrimental components, a score of 1 was assigned when participants’ intake was below the median or when their dietary fat constituted less than 40% of total energy intake; otherwise, a score of 0 was assigned. The individual scores were aggregated into two distinct sub-scores: the Beneficial Gut Microbiota Score (BGMS; ranging from 0 to 10) and the Unfavorable Gut Microbiota Score (UGMS; ranging from 0 to 4). The combined scores yielded a comprehensive DI-GM ranging from 0 to 14, with higher scores reflecting greater adherence to dietary patterns favorable to gut microbial health. Dietary intake was assessed based on the average of two validated 24-h dietary recalls—initially conducted in-person at Mobile Examination Centers (MEC) followed by a telephone-administered recall—to provide robust estimates of habitual dietary intake. Detailed scoring criteria and component-specific calculation methods are provided in Supplementary Table 1.

### 2.3 Periodontitis assessment

Periodontal assessments were rigorously performed by trained and calibrated dental examiners following standardized NHANES examination protocols. Participants aged 30 years or older with at least two natural teeth underwent full-mouth periodontal examinations at six specific sites per tooth (mesio-buccal, mid-buccal, disto-buccal, mesio-lingual, mid-lingual, and disto-lingual), excluding third molars. Probing depth (PD) and gingival recession were meticulously measured using a periodontal probe with 2-mm increments (HU-Friedy), and clinical attachment loss (AL) was calculated by summing PD and gingival recession at each site. These parameters are widely recognized markers of cumulative periodontal tissue destruction and inflammatory burden, and were selected for their established relevance in capturing the severity and extent of periodontitis (26).

Periodontitis cases were classified according to the Centers for Disease Control and Prevention–American Academy of Periodontology (CDC–AAP) surveillance criteria established by Eke et al. (27), categorizing periodontitis severity as mild (≥2 interproximal sites with AL ≥ 3 mm and ≥2 interproximal sites with PD ≥ 4 mm [non-adjacent], or ≥1 site with PD ≥ 5 mm), moderate (≥2 non-adjacent sites with AL ≥ 4 mm or PD ≥ 5 mm), and severe (≥2 non-adjacent sites with AL ≥ 6 mm and ≥1 site with PD ≥ 5 mm). Participants who did not meet criteria for moderate or severe periodontitis were grouped as having no or mild periodontitis. For analytical clarity and clinical relevance, periodontitis status was dichotomized into two categories—no/mild versus moderate/severe—which were subsequently used as binary outcomes in logistic regression analyses. This dichotomization aligns with common approaches in population-based epidemiological studies focusing on clinically significant periodontal disease progression (28, 29).

### 2.4 Assessment of inflammatory markers

Fasting venous blood samples were collected at NHANES Mobile Examination Centers following standardized protocols detailed in the NHANES Laboratory Procedures Manual. Serum C-reactive protein (CRP) was measured using latex-enhanced nephelometry (Behring Nephelometer II) exclusively during the 2009–2010 survey cycles. Additionally, complete blood counts—including total leukocytes, neutrophils, lymphocytes, and platelets—were analyzed with the Beckman Coulter DxH 800 hematology analyzer. Inflammatory biomarkers evaluated as potential mediators in the association between the Dietary Index for Gut Microbiota (DI-GM) and periodontitis included systemic inflammation markers (CRP, leukocyte count) and derived indices, such as neutrophil-to-lymphocyte ratio (NLR), platelet-to-lymphocyte ratio (PLR), and systemic immune-inflammation index (SII). These indices have been validated in prior research as comprehensive indicators of inflammatory status and are closely associated with periodontal disease pathogenesis (30–32). Given their skewed distributions, all biomarkers underwent log_2_ transformation to achieve approximate normality, facilitating subsequent statistical analyses.

### 2.5 Assessment of covariates

Covariates were selected based on previous evidence linking dietary patterns and oral health outcomes (33, 34). Data on these variables were collected through structured questionnaires, physical examinations, and laboratory tests during three consecutive NHANES survey cycles (2009–2014). Sociodemographic characteristics included age (continuous, in years), sex (male or female), race/ethnicity (non-Hispanic White, non-Hispanic Black, Mexican American, or other), educational attainment (<9th grade, 9th–11th grade, high school graduate, or college and above), and annual household income (categorized as ≥$20,000 or <$20,000). Lifestyle-related factors encompassed smoking status, alcohol consumption, physical activity, body mass index (BMI), and total daily energy intake. Smoking status was classified into never/former (lifetime consumption of ≥100 cigarettes but currently abstinent) and current smoker. Alcohol consumption was categorized as never drinker (<12 lifetime drinks), former drinker (≥12 drinks consumed in 1 year previously but no consumption in the past year, or lifetime consumption ≥12 drinks without recent use), current mild/moderate drinker (≤1 drink/day for women or ≤2 drinks/day for men), and current heavy drinker (>1 drink/day for women or >2 drinks/day for men) (35). Physical activity was quantified using metabolic equivalent task (MET)-minutes per week and subsequently stratified into tertiles. BMI was calculated using measured height and weight (kg/m^2^), and total daily energy intake (kcal/day) was estimated from the mean of two 24-h dietary recalls. Diabetes status was defined based on self-report of physician-diagnosed diabetes or biochemical confirmation via a hemoglobin A1c (HbA1c) level ≥6.5%.

### 2.6 Statistical analysis

Continuous variables were compared using analysis of variance (ANOVA), while categorical variables were analyzed using the *χ*^2^ test. Multivariable logistic regression models were employed to estimate odds ratios (ORs) and corresponding 95% confidence intervals (CIs) for associations between Dietary Index for Gut Microbiota (DI-GM) scores and periodontitis. Participants were categorized into four groups based on DI-GM scores: Q1 (0–4), Q2 (5), Q3 (6), and Q4 (7–14) (36). Model 1 was unadjusted, whereas Model 2 was adjusted for demographic factors including age, sex, and race/ethnicity. Model 3 additionally accounted for body mass index (BMI), educational attainment, annual household income, smoking status, alcohol consumption, diabetes status, physical activity, and total energy intake. Missing data (<25%) were addressed using multiple imputation techniques (five imputations), with integrated estimates derived through Rubin’s rules (37). Restricted cubic spline (RCS) models were utilized to investigate potential non-linear associations between DI-GM scores and periodontitis prevalence.

To further assess the association between DI-GM scores and the severity of periodontitis, we conducted multinomial logistic regression analyses with periodontitis severity categorized as no, mild, moderate, or severe (CDC–AAP definitions), using “no periodontitis” as the reference. The analysis was repeated for BGMS and UGMS. We selected multinomial rather than ordinal logistic regression, as the proportional odds assumption was not satisfied (Brant test *p* < 0.05). All models were adjusted for the same set of covariates as previously described.

To explore potential mechanisms underlying the observed association, we performed a mediation analysis focusing on systemic inflammatory markers. Mediation analyses were conducted using the R package “mediation” (bootstrap iterations = 5,000) to quantify the mediating role of systemic inflammation biomarkers [C-reactive protein (CRP), leukocyte count (WBC), neutrophil-to-lymphocyte ratio (NLR), platelet-to-lymphocyte ratio (PLR), and systemic immune-inflammation index (SII)] in the DI-GM–periodontitis relationship. Direct effects (DE) represented associations independent of mediators, whereas indirect effects (IE) captured associations mediated through biomarkers. The proportion mediated was calculated as IE divided by the total effect (TE).

Additionally, stratified and interaction analyses were conducted to investigate potential effect modifications across predefined subgroups. Multiple sensitivity analyses were performed to validate the robustness of our findings. First, alternative periodontal outcomes, including mean attachment loss (AL), mean probing pocket depth (PPD), and proportions of sites with PPD ≥ 4 mm or CAL ≥ 3 mm, were evaluated to provide a comprehensive assessment of periodontal health. Second, DI-GM scores were further categorized into tertiles and quintiles; tertile categorization (low/middle/high) addressed potential misclassification resulting from skewed DI-GM distributions, while quintile analysis allowed exploration of finer dose–response relationships and minimized residual confounding. Third, analyses were replicated on a complete-case dataset without imputation. Fourth, further adjustments were conducted to account for histories of hypertension, cardiovascular disease (CVD), and cancer to examine potential confounding from chronic diseases, and analyses were repeated after excluding participants with these conditions and diabetes.

All statistical analyses were performed using R version 4.4.2. The “mice” package (version 3.16.0) was utilized for multiple imputations, and mediation analysis was conducted with the “mediation” package (version 4.5.0). Statistical significance was defined as a two-tailed *p*-value <0.05.

## 3 Results

### 3.1 Baseline characteristics

The baseline characteristics of the study population stratified by periodontitis status are presented in Table 1. The analysis comprised 9,022 participants, of whom 48.6% were male. The overall mean age was 52.27 years, with a mean BMI of 29.45 kg/m^2^, and average total daily energy intake of 2041.4 kcal. Approximately 56.7% had completed education at the college graduate level or above, while 20.7% reported an annual household income below $20,000. The prevalence of current smoking, severe alcohol consumption, low physical activity, and diabetes was 17.8, 32.4, 34.4, and 15.8%, respectively. Compared with those without periodontitis, participants with periodontitis were significantly older, had higher BMI, lower Dietary Index for Gut Microbiota (DI-GM) scores, and were more likely to be male, non-white, less educated, of lower socioeconomic status, current smokers, and diabetic (all *p* < 0.001). Detailed characteristics stratified by DI-GM scores are provided in Supplementary Table 2.

**Table 1:** Baseline characteristics of study participants stratified by periodontitis status.

Characteristics	Total *n* = 9,022	Non-periodontitis *n* = 4,934 (54.69%)	Periodontitis *n* = 4,088 (45.31%)	*P*-value
Age, years	52.27 ± 14.19	48.61 ± 13.58	56.69 ± 13.65	<0.001
BMI, kg/m^2^	29.45 ± 6.73	29.29 ± 6.72	29.64 ± 6.74	0.01
DI-GM score	5.20 ± 1.75	5.34 ± 1.77	5.02 ± 1.71	<0.001
BGMS	2.59 ± 1.48	2.76 ± 1.48	2.39 ± 1.46	<0.001
UGMS	2.61 ± 1.06	2.59 ± 1.04	2.63 ± 1.07	0.04
Total energy intake, kcal	2041.40 ± 801.80	2049.39 ± 771.50	2031.76 ± 836.91	0.30
Sex				<0.001
Female	4,640 (51.43)	2,912 (59.02)	1728 (42.27)	
Male	4,382 (48.57)	2022 (40.98)	2,360 (57.73)	
Ethnicity				<0.001
Non-Hispanic White	4,060 (45.00)	2,491 (50.49)	1,569 (38.38)	
Non-Hispanic Black	1854 (20.55)	836 (16.94)	1,018 (24.90)	
Mexican American	1,262 (13.99)	555 (11.25)	707 (17.29)	
Other	1846 (20.46)	1,052 (21.32)	794 (19.42)	
Educational attainment				<0.001
Less than 9th grade	793(8.79)	266(5.39)	527 (12.89)	
9th–11th grade	1,165 (12.91)	453(9.18)	712 (17.42)	
High school grade	1946 (21.57)	908 (18.40)	1,038 (25.39)	
College graduate or above	5,118 (56.73)	3,307 (67.02)	1811 (44.30)	
Annual household income				<0.001
≥ $20,000	7,157 (79.33)	4,153 (84.17)	3,004 (73.48)	
< $20,000	1865 (20.67)	781 (15.83)	1,084 (26.52)	
Smoking status				<0.001
Never	5,087 (56.38)	3,191 (64.67)	1896 (46.38)	
Former	2,329 (25.81)	1,129 (22.88)	1,200 (29.35)	
Now	1,606 (17.80)	614 (12.44)	992 (24.27)	
Drinking status				<0.001
Never	1,183 (13.11)	616 (12.48)	567 (13.87)	
Former	1,592 (17.65)	692 (14.03)	900 (22.02)	
Mild/Moderate	3,321 (36.81)	1958 (39.68)	1,363 (33.34)	
Severe	2,926 (32.43)	1,668 (33.81)	1,258 (30.77)	
Diabetes status				<0.001
No	7,599 (84.23)	4,383 (88.83)	3,216 (78.67)	
Yes	1,423 (15.77)	551 (11.17)	872 (21.33)	
Physical activity				<0.001
Low	3,100 (34.36)	1712 (34.70)	1,388 (33.95)	
Moderate	2,942 (32.61)	1,689 (34.23)	1,253 (30.65)	
High	2,980 (33.03)	1,533 (31.07)	1,447 (35.40)	

BMI, body mass index; DI-GM score, dietary index for gut microbiota score; BGMS, beneficial to gut microbiota score; UGMS, unfavorable to gut microbiota score; NHANES, National Health and Nutrition Examination Survey. Continuous variables are presented as mean ± standard deviation (SD); categorical variables are reported as actual frequency (percentage [%]).

Missing data proportions are specified as follows: BMI (0.6%), educational attainment (0.1%), annual household income (3.7%), smoking status (0.04%), drinking status (4.9%), and physical activity (24.8%). No missing values were observed for age, sex, ethnicity, daily energy intake, or diabetes status.

### 3.2 Association between DI-GM and periodontitis

As shown in Table 2, higher DI-GM scores were significantly associated with lower prevalence of periodontitis across different adjustment models. In the unadjusted model (Model 1), each 1-point increase in DI-GM score corresponded to a 10% lower odds of periodontitis (OR: 0.90; 95% CI: 0.88–0.92; *p* < 0.001). This inverse association remained robust after adjustment for demographic characteristics including age, sex, and race/ethnicity (Model 2: OR: 0.89; 95% CI: 0.87–0.92; *p* < 0.001). After further comprehensive adjustment for socioeconomic factors (BMI, educational attainment, annual household income), behavioral factors (smoking status, alcohol consumption, physical activity), clinical conditions (diabetes status), and total energy intake (Model 3), the association persisted but was moderately attenuated (OR: 0.95; 95% CI: 0.92–0.97; *p* < 0.001).

**Table 2:** Associations between Dietary Index for Gut Microbiota (DI-GM) scores and periodontitis prevalence among NHANES 2009–2014 participants.

Characteristics	Model 1		Model 2		Model 3	
	OR (95% CI)	*P*-value	OR (95% CI)	*P*-value	OR (95% CI)	*P*-value
DI-GM group						
Q1	1(reference)		1(reference)		1(reference)	
Q2	0.81 (0.72,0.90)	<0.001	0.82 (0.73,0.93)	0.001	0.92 (0.81,1.04)	0.17
Q3	0.74 (0.66,0.83)	<0.001	0.71 (0.62,0.80)	<0.001	0.83 (0.73,0.95)	0.01
Q4	0.64 (0.57,0.72)	<0.001	0.62 (0.55,0.70)	<0.001	0.81 (0.71,0.92)	0.001
*P* for trend		<0.001		<0.001		<0.001
DI-GM score	0.9 (0.88,0.92)	<0.001	0.89 (0.87,0.92)	<0.001	0.95 (0.92,0.97)	<0.001
BGMS	0.84 (0.82,0.87)	<0.001	0.86 (0.83,0.89)	<0.001	0.91 (0.88,0.95)	<0.001
UGMS	1.04 (1.00,1.08)	0.04	0.98 (0.94,1.02)	0.36	1 (0.96,1.06)	0.85

BMI, body mass index; DI-GM, dietary index for gut microbiota; BGMS, beneficial to gut microbiota score; UGMS, unfavorable to gut microbiota score; NHANES, National Health and Nutrition Examination Survey; OR, odds ratio; CI, confidence interval.

Model 1 was not adjusted for any covariates.

Model 2 was additionally adjusted for age, sex, and race/ethnicity.

Model 3 was additionally adjusted for BMI, educational attainment, annual household income, smoking status, drinking status, diabetes status, physical activity and total energy intake.

Two-sided *P*-values are presented without adjustment for multiple comparisons, with *p*-values below 0.001 reported as <0.001.

When DI-GM scores were analyzed categorically, participants in higher DI-GM groups demonstrated progressively lower odds of periodontitis compared to those in the lowest DI-GM group. Specifically, participants in the highest DI-GM group exhibited significantly reduced odds of periodontitis in the fully adjusted model (Model 3: OR: 0.81; 95% CI: 0.71–0.92; *p* = 0.001), with a clear dose–response relationship observed across DI-GM groups (*P*_trend_ < 0.001). Additionally, each 1-point increment in the Beneficial Gut Microbiota Score (BGMS) was consistently associated with reduced odds of periodontitis in fully adjusted analyses (Model 3: OR: 0.91; 95% CI: 0.88–0.95; *p* < 0.001), whereas no significant association was detected between the Unfavorable Gut Microbiota Score (UGMS) and periodontitis after full adjustments (Model 3: OR: 1.00; 95% CI: 0.96–1.06; *p* = 0.85).

Moreover, when periodontitis severity was examined as a categorical outcome (no, mild, moderate, or severe), higher DI-GM scores were associated with progressively lower odds of moderate and severe periodontitis, but not with mild periodontitis. In fully adjusted models, individuals in the highest DI-GM group had 19% lower odds of moderate periodontitis (OR: 0.81; 95% CI: 0.79–0.83) and 26% lower odds of severe periodontitis (OR: 0.74; 95% CI: 0.71–0.76), compared with those in the lowest group. Additionally, for each 1-point increase in DI-GM score, the odds of moderate and severe periodontitis declined by 5% (OR: 0.95; 95% CI: 0.92–0.97) and 8% (OR: 0.92; 95% CI: 0.89–0.95), respectively. A similar trend was observed for BGMS, whereas UGMS remained unassociated with disease severity (Table 3).

**Table 3:** Associations between Dietary Index for Gut Microbiota (DI-GM) scores and severity of periodontitis among NHANES 2009–2014 participants.

Characteristics	Mild periodontitis	Moderate periodontitis	Severe periodontitis
	OR (95% CI)	OR (95% CI)	OR (95% CI)
DI-GM group			
Q1	1(reference)	1(reference)	1(reference)
Q2	1.18 (1.15, 1.2)	0.93 (0.93, 0.94)	0.93 (0.91, 0.96)
Q3	1.02 (0.99, 1.05)	0.86 (0.83, 0.89)	0.74 (0.73, 0.76)
Q4	0.81 (0.8, 0.82)	0.81 (0.79, 0.83)	0.74 (0.71, 0.76)
DI-GM score	0.98 (0.95, 1.00)	0.95 (0.92, 0.97)	0.92 (0.89, 0.95)
BGMS	0.96 (0.93, 0.99)	0.91 (0.89, 0.94)	0.89 (0.86, 0.93)
UGMS	1.01 (0.99, 1.03)	1.02 (0.99, 1.05)	0.96 (0.93, 1.00)

BMI, body mass index; DI-GM, dietary index for gut microbiota; BGMS, beneficial to gut microbiota score; UGMS, unfavorable to gut microbiota score; NHANES, National Health and Nutrition Examination Survey; OR, odds ratio; CI, confidence interval.

Models were fully adjusted for age, sex, race/ethnicity, BMI, educational attainment, annual household income, smoking status, drinking status, diabetes status, physical activity and total energy intake.

Two-sided *P*-values are presented without adjustment for multiple comparisons, with *p*-values below 0.001 reported as <0.001.

**Figure 1:**
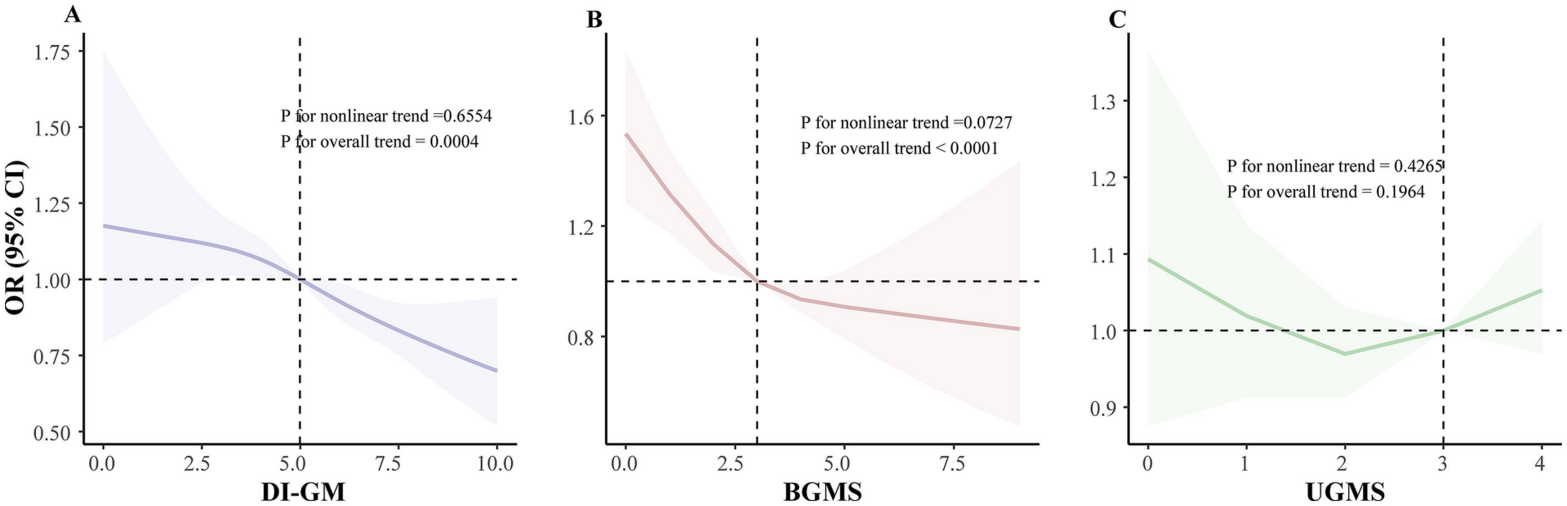
Dose–response relationship between DI-GM scores and periodontitis prevalence among NHANES 2009–2014 participants: restricted cubic spline analysis. **(A)** Linear association between DI-GM score and periodontitis prevalence. **(B)** Linear association between BGMS and periodontitis prevalence. **(C)** Linear association between UGMS and periodontitis prevalence. The model was adjusted for age, sex, race/ethnicity, BMI, educational attainment, annual household income, smoking status, drinking status, diabetes status, physical activity and total energy intake. The DI-GM score comprises BGMS and UGMS. BMI, body mass index; DI-GM, dietary index for gut microbiota; BGMS, beneficial to gut microbiota score; UGMS, unfavorable to gut microbiota score; NHANES, National Health and Nutrition Examination Survey; OR, odds ratio; CI, confidence interval; RCS, restricted cubic spline.

Finally, restricted cubic spline analyses provided additional evidence supporting linear inverse associations of DI-GM scores (*P*_non-linearity_ > 0.05), as well as BGMS, with the prevalence of periodontitis, further underscoring a consistent pattern where higher dietary quality beneficial to gut microbiota correlates with lower periodontitis prevalence (Figure 1).

### 3.3 Mediation analysis

**Figure 2:**
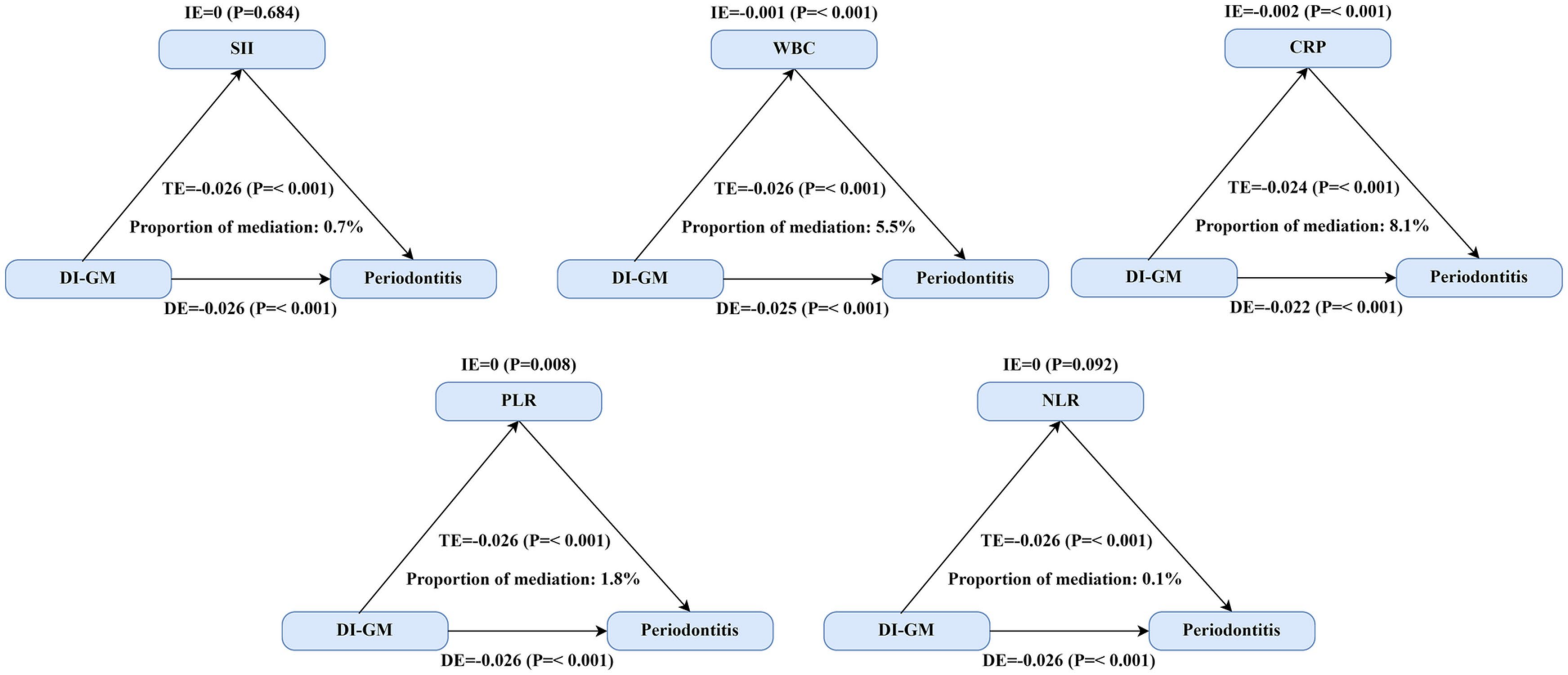
Mediating effect of systemic inflammation on the association between DI-GM scores and periodontitis. Three inflammatory biomarkers demonstrated significant mediation effects between DI-GM and periodontitis. The primary analysis included 8,773 participants for SII, WBC, PLR, and NLR analyses, while CRP analyses were restricted to 3,106 participants from the 2009–2010 NHANES cycle due to biomarker availability. TE = IE + DE; Proportion of mediation = IE/TE. Abbreviations: DI-GM, dietary index for gut microbiota; SII, systemic immune-inflammation index; PLR, platelet-to-lymphocyte ratio; NLR, neutrophil-to-lymphocyte ratio; WBC, white blood cell (leukocyte) count; CRP, C-reactive protein; DE, estimate of the direct effect; IE, estimate of the indirect effect; TE, estimate of the total effect.

We conducted mediation analyses to evaluate the extent to which systemic inflammation biomarkers mediated the relationship between the DI-GM and periodontitis. Significant mediation effects were identified for three biomarkers—leukocyte (WBC) count, platelet-to-lymphocyte ratio (PLR), and C-reactive protein (CRP)—while controlling for multiple sociodemographic, behavioral, and clinical covariates (Figure 2 and Table 4). Linear regression models demonstrated significant inverse associations between higher DI-GM scores and inflammation biomarkers after comprehensive covariate adjustments (Supplementary Table 4). Subsequently, elevated levels of systemic inflammatory markers, particularly CRP and WBC, were significantly associated with increased prevalence of periodontitis, whereas higher PLR levels correlated inversely with periodontitis prevalence (Supplementary Table 5). Specifically, CRP exhibited the highest proportion of mediation (8.1, 95% CI: 3.0–17.2%), followed by WBC (5.5, 95% CI: 3.2–8.6%) and PLR (1.8, 95% CI: 0.4–3.5%). In contrast, systemic immune-inflammation index (SII) and neutrophil-to-lymphocyte ratio (NLR) did not demonstrate significant mediation effects in the DI-GM-periodontitis association. Collectively, these findings suggest systemic inflammation partially mediates the beneficial impact of DI-GM on periodontal health.

**Table 4:** Mediation analysis evaluating the role of systemic inflammation biomarkers in the relationship between DI-GM scores and periodontitis.

Biomarkers	Total effect OR (95% CI)	Natural direct effect OR (95% CI)	Natural indirect effect OR (95% CI)	Percentage mediated % (95% CI)
SII	−0.026(−0.031, −0.021)	−0.026(−0.031, −0.02)	0 (0, −0.02)	0.001(−0.005, 0.007)
WBC (10^9^ cells/L)	−0.026(−0.032, −0.02)	−0.025(−0.03, −0.019)	−0.001(−0.002, −0.019)	0.055(0.032, 0.086)
PLR	−0.026(−0.032, −0.02)	−0.026(−0.031, −0.02)	0(−0.001, −0.02)	0.018(0.004, 0.035)
NLR	−0.026(−0.031, −0.02)	−0.026(−0.031, −0.02)	0(0, −0.02)	0.007(−0.003, 0.02)
CRP (mg/L)	−0.024(−0.033, −0.014)	−0.022(−0.031, −0.012)	−0.002(−0.003, −0.012)	0.081(0.03, 0.172)

DI-GM, dietary index for gut microbiota; SII, systemic immune-inflammation index; PLR, platelet-to-lymphocyte ratio; NLR, neutrophil-to-lymphocyte ratio; WBC, white blood cell (leukocyte) count; CRP, C-reactive protein; OR, odds ratio; CI, confidence interval.

Mediation analyses were adjusted for age, sex, race/ethnicity, BMI, educational attainment, annual household income, smoking status, drinking status, diabetes status, physical activity and total energy intake.

The levels of biomarkers were nature log-transformed before analyses. The proportion refers to per 1 score increment of DI-GM score.

### 3.4 Stratified and sensitivity analyses

**Figure 3:**
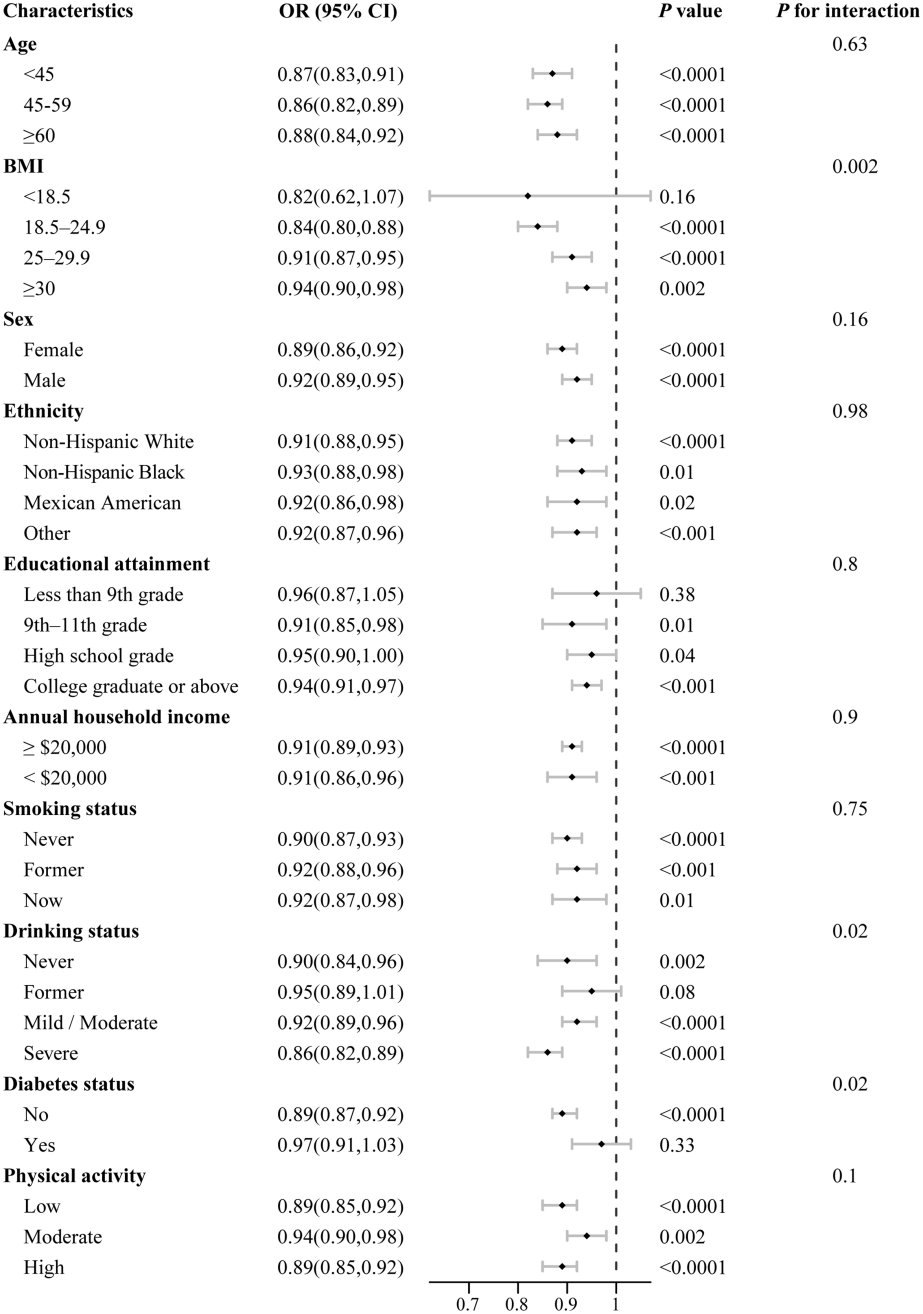
Stratified analysis of associations between DI-GM scores and periodontitis prevalence by selected demographic and clinical factors. All models were multivariable adjusted for age, sex, race/ethnicity, BMI, educational attainment, annual household income, smoking status, drinking status, diabetes status, physical activity and total energy intake. In each stratified analysis, the stratification variable was excluded in the adjustments. Data are presented as mean values (squares) and 95% CIs (error bars) for ORs. Likelihood ratio tests were used for assessment of interaction. Two-sided *p* values are presented without adjustment for multiple comparisons, with *p* values below 0.001 reported as <0.001.

We further conducted stratified analyses to evaluate the consistency of the association between DI-GM scores and the prevalence of periodontitis across subgroups defined by gender, age, race/ethnicity, BMI, educational attainment, annual household income, smoking status, drinking status, diabetes status, physical activity, and total energy intake (Figure 3). Generally, the inverse association between DI-GM scores and periodontitis was consistent across most subgroups, with no significant interactions identified for the majority of covariates (*P*_interaction_ > 0.05). However, interaction analyses indicated that the protective association of higher DI-GM scores against periodontitis varied significantly by age and smoking status (all *P*_interaction_ < 0.05), suggesting a potentially stronger protective effect among younger individuals and non-smokers.

Multiple sensitivity analyses consistently confirmed the robustness of our findings. When alternative periodontal health outcomes were assessed, the DI-GM score as a continuous variable exhibited significant inverse associations with mean attachment loss (AL: *β* = −0.02, 95% CI: −0.04 to −0.01), mean probing pocket depth (PPD: *β* = −0.02, 95% CI: −0.02 to −0.01), the proportion of sites with AL ≥ 3 mm (*β* = −0.38, 95% CI: −0.57 to −0.19), and the proportion of sites with PPD ≥ 4 mm (*β* = −0.14, 95% CI: −0.24 to −0.04). Similar inverse relationships were consistently observed when participants were categorized by DI-GM groups (Supplementary Tables 6–9). When DI-GM scores were further categorized by tertiles or quintiles, significant inverse associations with periodontitis prevalence persisted, demonstrating clear dose–response trends across groups (tertiles: OR_T3 versus T1_ = 0.81, 95% CI: 0.71–0.92; quintiles: OR_Q5 versus Q1_ = 0.77, 95% CI: 0.66–0.90) (Supplementary Tables 10, 11). Moreover, sensitivity analyses using complete-case data yielded comparable results to primary analyses (OR_Q4 versus Q1_ = 0.78, 95% CI: 0.67–0.92) (Supplementary Table 12). Additional adjustment for baseline histories of chronic diseases, including hypertension, cardiovascular disease, and cancer, did not materially alter these associations (OR_Q4 versus Q1_ = 0.81, 95% CI: 0.71–0.92), and similar results were observed after excluding participants with these chronic conditions and diabetes (OR_Q4 versus Q1_ = 0.76, 95% CI: 0.63–0.92) (Supplementary Tables 13, 14). Collectively, these comprehensive sensitivity analyses reinforce the reliability and stability of our primary findings.

## 4 Discussion

In this nationally representative cross-sectional study of U. S. adults, we identified a significant inverse association between adherence to the Dietary Index for Gut Microbiota (DI-GM) and periodontitis prevalence. Specifically, participants in the highest DI-GM group demonstrated a 19% lower odds of periodontitis compared with those in the lowest group. Furthermore, each incremental increase in DI-GM score was associated with a 5% reduction in periodontitis prevalence, reflecting a clear dose–response relationship. Mediation analyses indicated that systemic inflammation biomarkers, particularly C-reactive protein (CRP) and white blood cell (WBC) count, modestly but significantly mediated between 5.5 and 8.1% of the association. These findings, to our knowledge, are the first to substantiate the role of a dietary pattern beneficial to gut microbiota in periodontal health, introducing the novel concept of a ‘gut-oral axis’ regulated by dietary habits. This expands the conventional nutritional perspective beyond localized periodontal care, suggesting systemic microbial-immune modulation as a promising target for preventive dietary strategies against periodontal disease.

Our study provides robust epidemiological evidence linking higher adherence to the DI-GM with lower prevalence of periodontitis, thereby establishing DI-GM as the first dietary index explicitly connecting gut microbial modulation with oral inflammatory outcomes. This finding substantially extends prior dietary pattern studies, which have consistently reported inverse associations between periodontitis and dietary quality measures, including plant-based diets [OR = 0.93; Li et al. (28)], Mediterranean dietary patterns [pooled OR = 0.96; Fan et al. (38)], and the Healthy Eating Index [OR = 0.69; Li et al. (39)]. Although these established indices broadly capture dietary quality, DI-GM specifically prioritizes foods known to influence gut microbiota composition and activity (14), thus providing more precise biological insights into the diet-periodontitis relationship. For example, DI-GM uniquely emphasizes fermented foods and polyphenol-rich items (e.g., coffee and berries) demonstrated to enhance periodontal-protective bacterial taxa, such as *Akkermansia muciniphila* and *Faecalibacterium prausnitzii* (40–42). This biological specificity may explain why DI-GM retains predictive validity even when moderately correlated with Healthy Eating Index-2015 (*r* = 0.54) and Mediterranean Diet Score (*r* = 0.42) (14). Unlike traditional dietary indices that reflect general nutritional adequacy, DI-GM operationalizes a microbiome-focused dietary strategy, potentially explaining its value in predicting microbiota-associated inflammatory diseases, including periodontitis.

Biologically, the observed association between DI-GM and reduced periodontitis risk may be plausibly linked to diet-induced immunometabolic changes. While gut microbiota profiles were not directly measured in our study, previous experimental research suggests that microbial metabolites, particularly short-chain fatty acids (SCFAs), possess systemic anti-inflammatory and immune-regulatory properties (43, 44). Experimental studies consistently demonstrate that SCFAs, especially butyrate, enhance mucosal barrier function, suppress NF-κB-mediated inflammatory responses in gingival fibroblasts, and inhibit periodontal pathogen proliferation and biofilm formation (45–48). In contrast, dietary patterns characterized by high saturated fat and refined carbohydrate intake—negatively weighted within the DI-GM—have been associated with gut dysbiosis and elevated endotoxemia, which may exacerbate periodontal inflammation via TLR4/MyD88 pathways (49, 50). Although speculative in the absence of microbiota sequencing data, our findings are consistent with prior epidemiological observations. For instance, a Danish population-based study found that fermented dairy intake—a positively weighted DI-GM component—was inversely associated with periodontitis incidence (IRR = 0.97) (51). Overall, these results support the utility of DI-GM as a hypothesis-generating tool for identifying dietary patterns potentially relevant to inflammatory oral diseases. Further studies integrating dietary, microbiome, and immunologic data are warranted to delineate the causal pathways underlying these associations.

In addition to confirming the association between the DI-GM and periodontitis prevalence, our study further explored the potential inflammatory pathways mediating this relationship. Specifically, our mediation analysis highlighted the pivotal roles of systemic inflammatory biomarkers, particularly CRP and WBC count, in explaining the inverse association between DI-GM scores and periodontitis prevalence.

Inflammation is a critical pathological mechanism in the development and progression of periodontitis (52). Among systemic inflammatory markers, CRP has been consistently validated as a sensitive biomarker of host inflammatory burden and is closely linked to periodontal tissue degradation (22, 53). Previous studies have shown that pro-inflammatory dietary patterns—such as those high in refined carbohydrates—are significantly associated with elevated serum CRP levels, which in turn contribute to periodontal attachment loss and increased risk of periodontitis (54, 55). Our findings extend these observations, demonstrating that lower DI-GM scores, are positively correlated with both higher CRP concentrations and increased periodontitis prevalence. Notably, while CRP accounted for the largest proportion of mediation among the inflammatory biomarkers examined, it explained only 8.1% of the total association between DI-GM and periodontitis, suggesting a modest contribution. Several biological mechanisms may explain this observation. First, gut microbiota-derived metabolites, particularly lipopolysaccharides (LPS), can translocate across a compromised intestinal barrier and stimulate hepatic CRP synthesis via activation of toll-like receptor 4 (TLR4)-mediated pathways (56, 57). Elevated CRP, in turn, may contribute to periodontal pathology by amplifying systemic inflammatory tone and enhancing neutrophil infiltration into gingival tissues (58). Additionally, gut dysbiosis associated with low DI-GM scores may promote systemic low-grade inflammation, leading to a heightened inflammatory milieu that exacerbates local immune responses and promotes tissue degradation within the periodontium (59, 60). While other markers of systemic inflammation, including white blood cell (WBC) count and the platelet-to-lymphocyte ratio (PLR), demonstrated partial mediation effects (5.5 and 1.8%, respectively), their explanatory power was notably lower than that of CRP. Collectively, these findings support the hypothesis that systemic inflammation—particularly as mediated by CRP—is a critical mechanistic pathway through which gut microbiota-targeted dietary patterns influence the onset and progression of periodontitis.

The strengths should also be emphasized. This is the first population-based study to explore the role of systemic inflammation in mediating the relationship between DI-GM scores and periodontitis prevalence, offering novel insights into mechanistic pathways. Additionally, the use of NHANES 2009–2014 data ensures a large, nationally representative sample with rigorous protocols, enhancing the generalizability of our findings. However, several limitations of this study must be acknowledged. First, due to the cross-sectional nature of the data, we cannot infer causality between DI-GM scores and periodontitis prevalence. Temporal ambiguity exists, as reverse causality cannot be excluded. Additionally, mediation analysis in cross-sectional data may overestimate mediation effects, as exposure, mediator, and outcome are measured concurrently. Longitudinal studies are needed to confirm the causal relationship between diet and periodontal disease. Second, dietary measurements relied on self-reported 24-h dietary recalls, which are subject to recall bias and underreporting, particularly for high-fat foods. While these data provide valuable insights into dietary patterns at the time of data collection, they may not fully reflect long-term dietary habits, which could introduce measurement error in estimating the relationship between DI-GM and periodontitis. Third, the classification of periodontitis into dichotomous categories (no/mild vs. moderate/severe) may obscure important disease severity gradients. To address this, we performed additional multinomial and sensitivity analyses using the full CDC–AAP classification and alternative periodontal measures, offering more granular insights and reinforcing the robustness of our findings. Lastly, residual confounding from unmeasured factors, such as genetic predispositions, oral hygiene habits, or unaccounted-for dietary components, remains a possibility. Despite these limitations, the large sample size, rigorous data collection methods, and sensitivity analyses provide confidence in the reliability of our findings.

## 5 Conclusion

This nationally representative study found that greater adherence to the DI-GM was associated with lower periodontitis prevalence, particularly moderate and severe forms, partly mediated by systemic inflammation. These findings suggest that microbiota-targeted dietary patterns may help prevent both the onset and progression of periodontal disease and support the need for prospective, microbiome-informed research and policy-focused nutritional guidelines.

## Data Availability

The raw data supporting the conclusions of this article will be made available by the authors, without undue reservation.
