# Supplementary material for: Association between the Dietary Index for Gut Microbiota and periodontitis: mediation by systemic inflammation
Source: Front Nutr. 2025 Aug 13;12:1612199. doi: 10.3389/fnut.2025.1612199 (PMC12380554; doi:10.3389/fnut.2025.1612199)
Supplement: Supplementary file 1 [file Table_1.DOCX]

**Supplementary Materials**

**Supplementary Figure 1.** Flowchart of participant selection from NHANES 2009–2014.

**Supplementary Table 1.** Components and scoring criteria of the Dietary Index for Gut Microbiota (DI-GM).

**Supplementary Table 2.** Participant characteristics stratified by DI-GM score.

**Supplementary Table 3.** Characteristics of participants included in the mediation analysis.

**Supplementary Table 4.** Multivariable-adjusted associations between DI-GM scores and systemic inflammation biomarkers.

**Supplementary Table 5.** Associations between systemic inflammation biomarkers and periodontitis prevalence.

**Supplementary Table 6.** Associations between DI-GM scores and mean attachment loss (AL) among NHANES 2009–2014 participants.

**Supplementary Table 7.** Associations between DI-GM scores and mean probing pocket depth (PPD) among NHANES 2009–2014 participants.

**Supplementary Table 8.** Associations between DI-GM scores and the proportion of sites with attachment loss (AL) ≥ 3 mm among NHANES 2009–2014 participants.

**Supplementary Table 9.** Associations between DI-GM scores and the proportion of sites with probing pocket depth (PPD) ≥ 4 mm among NHANES 2009–2014 participants.

**Supplementary Table 10. Sensitivity analysis evaluating associations between DI-GM scores (categorized by tertiles) and periodontitis prevalence among NHANES 2009–2014 participants.**

**Supplementary Table 11. Additional sensitivity analysis assessing associations between DI-GM scores (categorized by quintiles) and periodontitis prevalence among NHANES 2009–2014 participants.**

**Supplementary Table 12.** Sensitivity analysis using complete-case data for associations between DI-GM scores and periodontitis prevalence among NHANES 2009–2014 participants.

**Supplementary Table 13.** Sensitivity analysis of associations between DI-GM scores and periodontitis prevalence after adjustment for major chronic diseases.

**Supplementary Table 14.** Sensitivity analysis of associations between DI-GM scores and periodontitis prevalence after excluding participants with chronic diseases (N=4,474).

****
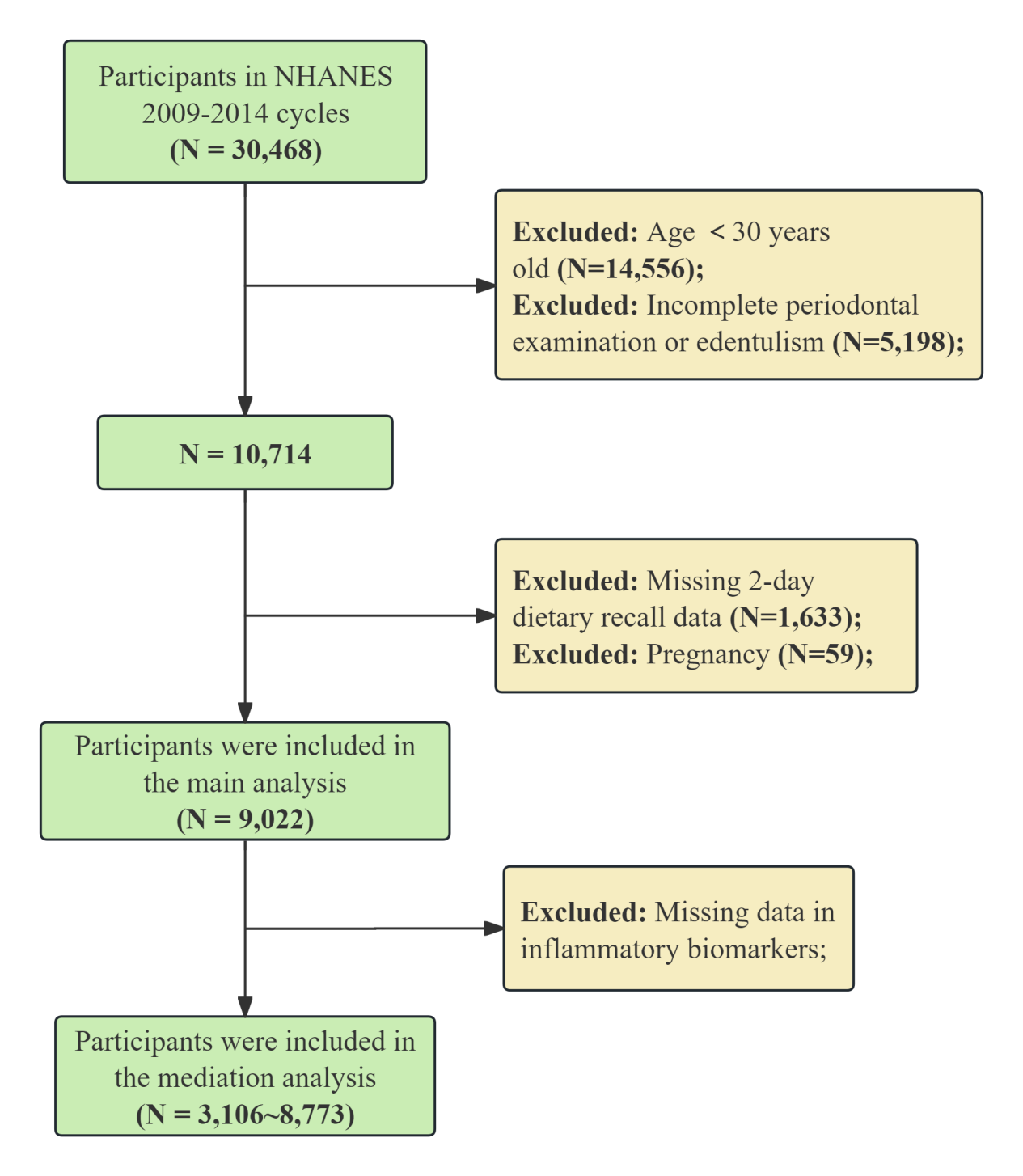
****

**Supplementary Figure 1.** Flowchart of participant selection from NHANES 2009–2014.

Missing data in platelet-to-lymphocyte ratio (PLR), neutrophil-to-lymphocyte ratio (NLR), systemic immune-inflammation index (SII), or leukocyte count (WBC) led to exclusion of 249 participants. However, C-reactive protein measurements were exclusively available in the 2009-2010 NHANES cycle per CDC laboratory protocols, resulting in 5,667 exclusions.

**Supplementary Table 1.** Components and scoring criteria of the Dietary Index for Gut Microbiota (DI-GM).

| **Component** | **Included Foods** | **Scoring criteria** |
| --- | --- | --- |
| **Beneficial to gut microbiota** | Avocados | Score 1 - Consumption≥sex-specific median  Score 0 - Otherwise |
|  | Broccoli |  |
|  | Chickpeas |  |
|  | Coffee |  |
|  | Cranberries |  |
|  | Fermented dairy (including yogurt, cheese, kefir, sour cream, buttermilk) |  |
|  | Fiber |  |
|  | Soybean (including Soy milk, Tofu) |  |
|  | Whole grains (containing the entire grain kernel—the bran, germ, and endosperm) |  |
|  | Green tea |  |
| **Unfavorable to gut microbiota** | Refined grains (Refined grains that do not contain all of the components of the entire grain kernel) | Score 0 - Consumption≥sex-specific median  Score 1 - Otherwise |
|  | Processed meat (Frankfurters, sausages, corned beef, and luncheon meat that are made from beef, pork, or poultry) |  |
|  | Red meat (Beef, veal, pork, lamb, and game meat; excludes organ meat and cured meat) |  |
|  | High-fat diet (% energy) | Score 0 - Consumption≥40%  Score 1 - Otherwise |

Abbreviations: DI-GM, dietary index for gut microbiota; NHANES, National Health and Nutrition Examination Survey.

**Supplementary Table 2.** Participant characteristics stratified by DI-GM score.

| **Characteristics** | **Total (*n* = 9,022)** | **Q1 (*n*= 3,221)** | **Q2 (*n*= 2,015)** | **Q3 (*n*= 1,727)** | **Q4 (*n*= 2,059)** | ***P* value** |
| --- | --- | --- | --- | --- | --- | --- |
| **Age, years** | 52.27 ± 14.19 | 51.29 ± 14.07 | 51.64 ± 13.94 | 52.83 ± 14.57 | 53.97 ± 14.14 | <0.001 |
| **BMI, kg/m^2^** | 29.45 ± 6.73 | 30.02 ± 7.04 | 29.84 ± 6.79 | 29.22 ± 6.37 | 28.37 ± 6.32 | <0.001 |
| **Total energy intake, kcal** | 2041.40 ± 801.80 | 2024.65 ± 825.24 | 2050.70 ± 855.26 | 2025.71 ± 770.77 | 2071.69 ± 732.63 | 0.15 |
| **Periodontitis status** |  |  |  |  |  | <0.001 |
| No | 4934(54.69) | 1597(49.58) | 1106(54.89) | 985(57.04) | 1246(60.51) |  |
| Yes | 4088(45.31) | 1624(50.42) | 909(45.11) | 742(42.96) | 813(39.49) |  |
| **Sex** |  |  |  |  |  | <0.001 |
| Female | 4640(51.43) | 1570(48.74) | 1049(52.06) | 874(50.61) | 1147(55.71) |  |
| Male | 4382(48.57) | 1651(51.26) | 966(47.94) | 853(49.39) | 912(44.29) |  |
| **Ethnicity** |  |  |  |  |  | <0.001 |
| Non-Hispanic White | 4060(45.00) | 1281(39.77) | 903(44.81) | 821(47.54) | 1055(51.24) |  |
| Non-Hispanic Black | 1854(20.55) | 839(26.05) | 414(20.55) | 308(17.83) | 293(14.23) |  |
| Mexican American | 1262(13.99) | 490(15.21) | 318(15.78) | 244(14.13) | 210(10.20) |  |
| Other | 1846(20.46) | 611(18.97) | 380(18.86) | 354(20.50) | 501(24.33) |  |
| **Educational attainment** |  |  |  |  |  | <0.001 |
| Less than 9th grade | 793( 8.79) | 341(10.59) | 180( 8.93) | 163( 9.44) | 109( 5.29) |  |
| 9th–11th grade | 1165(12.91) | 529(16.42) | 269(13.35) | 185(10.71) | 182( 8.84) |  |
| High school grade | 1946(21.57) | 839(26.05) | 435(21.59) | 329(19.05) | 343(16.66) |  |
| College graduate or above | 5118(56.73) | 1512(46.94) | 1131(56.13) | 1050(60.80) | 1425(69.21) |  |
| **Annual household income** |  |  |  |  |  | <0.001 |
| ≥ $20,000 | 7157(79.33) | 2433(75.54) | 1593(79.06) | 1384(80.14) | 1747(84.85) |  |
| < $20,000 | 1865(20.67) | 788(24.46) | 422(20.94) | 343(19.86) | 312(15.15) |  |
| **Smoking status** |  |  |  |  |  | <0.001 |
| Never | 5087(56.38) | 1725(53.55) | 1136(56.38) | 1025(59.35) | 1201(58.33) |  |
| Former | 2329(25.81) | 761(23.63) | 517(25.66) | 439(25.42) | 612(29.72) |  |
| Now | 1606(17.80) | 735(22.82) | 362(17.97) | 263(15.23) | 246(11.95) |  |
| **Drinking status** |  |  |  |  |  | <0.001 |
| Never | 1183(13.11) | 424(13.16) | 269(13.35) | 220(12.74) | 270(13.11) |  |
| Former | 1592(17.65) | 596(18.50) | 358(17.77) | 327(18.93) | 311(15.10) |  |
| Mild/Moderate | 3321(36.81) | 1058(32.85) | 705(34.99) | 657(38.04) | 901(43.76) |  |
| Severe | 2926(32.43) | 1143(35.49) | 683(33.90) | 523(30.28) | 577(28.02) |  |
| **Diabetes status** |  |  |  |  |  | <0.01 |
| No | 7599(84.23) | 2663(82.68) | 1691(83.92) | 1465(84.83) | 1780(86.45) |  |
| Yes | 1423(15.77) | 558(17.32) | 324(16.08) | 262(15.17) | 279(13.55) |  |
| **Physical activity** |  |  |  |  |  | <0.001 |
| Low | 3100(34.36) | 1084(33.65) | 691(34.29) | 620(35.90) | 705(34.24) |  |
| Moderate | 2942(32.61) | 951(29.52) | 668(33.15) | 581(33.64) | 742(36.04) |  |
| High | 2980(33.03) | 1186(36.82) | 656(32.56) | 526(30.46) | 612(29.72) |  |

Abbreviations: BMI, body mass index; DI-GM score, dietary index for gut microbiota score; BGMS, beneficial to gut microbiota score; UGMS, unfavorable to gut microbiota score; NHANES, National Health and Nutrition Examination Survey.

Continuous variables are presented as mean ± standard deviation (SD); categorical variables are reported as actual frequency (percentage [%]).

Missing data proportions are specified as follows: BMI (0.6%), educational attainment (0.1%), annual household income (3.7%), smoking status (0.04%), drinking status (4.9%), and physical activity (24.8%). No missing values were observed for age, sex, ethnicity, daily energy intake, or diabetes status.

**Supplementary Table 4.** Multivariable-adjusted associations between DI-GM scores and systemic inflammation biomarkers.

| Characteristics | Model 1 | | Model 2 | | Model 3 | |
| --- | --- | --- | --- | --- | --- | --- |
|  | *β* (95% CI) | *P* value | *β* (95% CI) | *P* value | *β* (95% CI) | *P* value |
| SII | -0.01(-0.01,0.00) | 0.05 | -0.02(-0.02,-0.01) | <0.001 | -0.01(-0.02, 0.00) | <0.001 |
| PLR | 0.01(0.00,0.01) | 0.01 | 0( 0.00, 0.01) | 0.25 | 0(-0.01, 0.00) | 0.30 |
| NLR | 0(-0.01,0.00) | 0.15 | -0.01(-0.02,-0.01) | <0.001 | -0.01(-0.02, 0.00) | <0.001 |
| WBC (10^9^ cells/L) | -0.01(-0.02,-0.01) | <0.001 | -0.01(-0.02,-0.01) | <0.001 | -0.01(-0.01, 0.00) | <0.001 |
| CRP (mg/L) | -0.08(-0.10,-0.05) | <0.001 | -0.07(-0.10,-0.05) | <0.001 | -0.04(-0.06,-0.01) | 0.001 |

Abbreviations: DI-GM, dietary index for gut microbiota; SII, systemic immune-inflammation index; PLR, platelet-to-lymphocyte ratio; NLR, neutrophil-to-lymphocyte ratio; WBC, white blood cell (leukocyte) count; CRP, C-reactive protein; CI, confidence interval.

Model 1 was not adjusted for any covariates.

Model 2 was adjusted for age, sex, and race/ethnicity.

Model 3 was additionally adjusted for BMI, educational attainment, annual household income, smoking status, drinking status, diabetes status, physical activity and total energy intake.

**Supplementary Table 5.** Associations between systemic inflammation biomarkers and periodontitis prevalence.

| Characteristics | Model 1 | | Model 2 | | Model 3 | |
| --- | --- | --- | --- | --- | --- | --- |
|  | OR (95% CI) | *P* value | OR (95% CI) | *P* value | OR (95% CI) | *P* value |
| **SII** |  |  |  |  |  |  |
| Tertile 1 | 1(reference) |  | 1(reference) |  | 1(reference) |  |
| Tertile 2 | 0.88(0.79,0.97) | 0.01 | 1.02(0.91,1.14) | 0.70 | 1.01(0.90,1.14) | 0.84 |
| Tertile 3 | 1(0.91,1.11) | 0.95 | 1.2(1.08,1.35) | 0.001 | 1.11(0.98,1.25) | 0.10 |
| P for trend |  | 0.95 |  | 0.001 |  | 0.1 |
| Per standard  deviation increment | 1.01(0.97,1.06) | 0.56 | 1.09(1.04,1.14) | <0.001 | 1.05(1.00,1.11) | 0.04 |
| **WBC** |  |  |  |  |  |  |
| Tertile 1 | 1(reference) |  | 1(reference) |  | 1(reference) |  |
| Tertile 2 | 1.08(0.97,1.20) | 0.14 | 1.18(1.05,1.32) | 0.01 | 1.06(0.94,1.20) | 0.31 |
| Tertile 3 | 1.38(1.25,1.53) | <0.001 | 1.81(1.62,2.03) | <0.001 | 1.4(1.24,1.58) | <0.0001 |
| P for trend |  | <0.001 |  | <0.001 |  | <0.0001 |
| Per standard  deviation increment | 1.17(1.12,1.22) | <0.001 | 1.33(1.27,1.40) | <0.001 | 1.17(1.11,1.24) | <0.0001 |
| **PLR** |  |  |  |  |  |  |
| Tertile 1 | 1(reference) |  | 1(reference) |  | 1(reference) |  |
| Tertile 2 | 0.81(0.73,0.89) | <0.001 | 0.85(0.76,0.95) | 0.003 | 0.95(0.85,1.07) | 0.43 |
| Tertile 3 | 0.75(0.68,0.83) | <0.001 | 0.75(0.67,0.84) | <0.001 | 0.89(0.79,1.00) | 0.05 |
| P for trend |  | <0.001 |  | <0.001 |  | 0.05 |
| Per standard  deviation increment | 0.89(0.85,0.93) | <0.001 | 0.88(0.84,0.92) | <0.001 | 0.94(0.90,0.99) | 0.02 |
| **NLR** |  |  |  |  |  |  |
| Tertile 1 | 1(reference) |  | 1(reference) |  | 1(reference) |  |
| Tertile 2 | 0.92(0.83,1.02) | 0.10 | 0.97(0.86,1.08) | 0.55 | 0.96(0.85,1.08) | 0.47 |
| Tertile 3 | 1.16(1.04,1.28) | 0.01 | 1.13(1.01,1.27) | 0.03 | 1.05(0.93,1.18) | 0.47 |
| P for trend |  | 0.01 |  | 0.03 |  | 0.47 |
| Per standard  deviation increment | 1.1(1.05,1.14) | <0.001 | 1.09(1.04,1.14) | <0.001 | 1.06(1.01,1.11) | 0.02 |
| **CRP** |  |  |  |  |  |  |
| Tertile 1 | 1(reference) |  | 1(reference) |  | 1(reference) |  |
| Tertile 2 | 1.24(1.04,1.48) | 0.01 | 1.13(0.93,1.37) | 0.21 | 1.04(0.85,1.28) | 0.70 |
| Tertile 3 | 1.25(1.05,1.48) | 0.01 | 1.27(1.05,1.54) | 0.01 | 1.09(0.87,1.36) | 0.46 |
| P for trend |  | 0.01 |  | 0.01 |  | 0.46 |
| Per standard  deviation increment | 1.16(1.08,1.24) | <0.001 | 1.15(1.06,1.25) | <0.001 | 1.08(0.98,1.19) | 0.10 |

Abbreviations: SII, systemic immune-inflammation index; PLR, platelet-to-lymphocyte ratio; NLR, neutrophil-to-lymphocyte ratio; WBC, white blood cell (leukocyte) count; CRP, C-reactive protein; OR, odds ratio; CI, confidence interval.

Model 1 was not adjusted for any covariates.

Model 2 was adjusted for age, sex, and race/ethnicity.

Model 3 was additionally adjusted for BMI, educational attainment, annual household income, smoking status, drinking status, diabetes status, physical activity and total energy intake.

**Supplementary Table 6.** Associations between DI-GM scores and mean clinical attachment loss (CAL) among NHANES 2009–2014 participants.

| Characteristics | Model 1 | | Model 2 | | Model 3 | |
| --- | --- | --- | --- | --- | --- | --- |
|  | *β* (95% CI) | *P* value | *β* (95% CI) | *P* value | *β* (95% CI) | *P* value |
| DI-GM group |  |  |  |  |  |  |
| Q1 | 1(reference) |  | 1(reference) |  | 1(reference) |  |
| Q2 | -0.09(-0.16,-0.03) | 0.01 | -0.06(-0.12, 0.00) | 0.06 | 0.02(-0.04, 0.07) | 0.60 |
| Q3 | -0.21(-0.28,-0.14) | <0.001 | -0.2(-0.26,-0.13) | <0.001 | -0.09(-0.15,-0.03) | 0.005 |
| Q4 | -0.28(-0.34,-0.21) | <0.001 | -0.24(-0.30,-0.18) | <0.001 | -0.07(-0.13,-0.01) | 0.02 |
| P for trend |  | <0.001 |  | <0.001 |  | 0.002 |
| DI-GM score | -0.07(-0.08,-0.06) | <0.001 | -0.06(-0.07,-0.05) | <0.001 | -0.02(-0.04,-0.01) | <0.001 |
| BGMS | -0.11(-0.12,-0.09) | <0.001 | -0.08(-0.09,-0.06) | <0.001 | -0.03(-0.05,-0.02) | <0.001 |
| UGMS | 0.02(-0.01,0.04) | 0.18 | -0.01(-0.03,0.01) | 0.29 | -0.01(-0.03, 0.01) | 0.40 |

**Abbreviation:** DI-GM, dietary index for gut microbiota; CAL, clinical attachment loss; BGMS, beneficial to gut microbiota score; UGMS, unfavorable to gut microbiota score; NHANES, National Health and Nutrition Examination Survey; OR, odds ratio; CI, confidence interval; BMI, body mass index.

Model 1 was not adjusted for any covariates.

Model 2 was adjusted for age, sex, and race/ethnicity.

Model 3 was additionally adjusted for BMI, educational attainment, annual household income, smoking status, drinking status, diabetes status, physical activity and total energy intake.

Two-sided *P* values are presented without adjustment for multiple comparisons, with *P* values below 0.001 reported as <0.001.

**Supplementary Table 7.** Associations between DI-GM scores and mean probing pocket depth (PPD) among NHANES 2009–2014 participants.

| Characteristics | Model 1 | | Model 2 | | Model 3 | |
| --- | --- | --- | --- | --- | --- | --- |
|  | *β* (95% CI) | *P* value | *β* (95% CI) | *P* value | *β* (95% CI) | *P* value |
| DI-GM group |  |  |  |  |  |  |
| Q1 | 1(reference) |  | 1(reference) |  | 1(reference) |  |
| Q2 | -0.08(-0.12,-0.04) | <0.001 | -0.06(-0.09,-0.02) | 0.002 | -0.02(-0.05, 0.02) | 0.27 |
| Q3 | -0.15(-0.19,-0.11) | <0.001 | -0.12(-0.16,-0.08) | <0.001 | -0.06(-0.10,-0.02) | 0.001 |
| Q4 | -0.22(-0.26,-0.18) | <0.001 | -0.15(-0.19,-0.12) | <0.001 | -0.06(-0.10,-0.02) | <0.001 |
| P for trend |  | <0.001 |  | <0.001 |  | <0.001 |
| DI-GM score | -0.05(-0.06,-0.04) | <0.001 | -0.04(-0.05,-0.03) | <0.001 | -0.02(-0.02,-0.01) | <0.001 |
| BGMS | -0.06(-0.07,-0.05) | <0.001 | -0.05(-0.06,-0.04) | <0.001 | -0.02(-0.03,-0.01) | <0.001 |
| UGMS | -0.02(-0.04,-0.01) | 0.001 | -0.01(-0.03,0.00) | 0.05 | -0.01(-0.02, 0.01) | 0.31 |

**Abbreviation:** DI-GM, dietary index for gut microbiota; PPD: probing pocket depth; BGMS, beneficial to gut microbiota score; UGMS, unfavorable to gut microbiota score; NHANES, National Health and Nutrition Examination Survey; OR, odds ratio; CI, confidence interval; BMI, body mass index.

Model 1 was not adjusted for any covariates.

Model 2 was adjusted for age, sex, and race/ethnicity.

Model 3 was additionally adjusted for BMI, educational attainment, annual household income, smoking status, drinking status, diabetes status, physical activity and total energy intake.

Two-sided *P* values are presented without adjustment for multiple comparisons, with *P* values below 0.001 reported as <0.001.

**Supplementary Table 8.** Associations between DI-GM scores and the proportion of sites with clinical attachment loss (CAL) ≥ 3 mm among NHANES 2009–2014 participants.

| Characteristics | Model 1 | | Model 2 | | Model 3 | |
| --- | --- | --- | --- | --- | --- | --- |
|  | *β* (95% CI) | *P* value | *β* (95% CI) | *P* value | *β* (95% CI) | *P* value |
| DI-GM group |  |  |  |  |  |  |
| Q1 | 1(reference) |  | 1(reference) |  | 1(reference) |  |
| Q2 | -1.19(-2.13,-0.25) | 0.01 | -0.79(-1.69, 0.11) | 0.08 | -0.24(-1.11, 0.63) | 0.59 |
| Q3 | -2.12(-3.11,-1.14) | <0.001 | -1.86(-2.80,-0.91) | <0.001 | -0.97(-1.89,-0.05) | 0.04 |
| Q4 | -3.36(-4.29,-2.42) | <0.001 | -2.64(-3.54,-1.74) | <0.001 | -1.34(-2.23,-0.46) | 0.003 |
| P for trend |  | <0.001 |  | <0.001 |  | 0.001 |
| DI-GM score | -0.83(-1.03,-0.63) | <0.001 | -0.68(-0.87,-0.48) | <0.001 | -0.38(-0.57,-0.19) | <0.001 |
| BGMS | -1.18(-1.41,-0.94) | <0.001 | -0.88(-1.10,-0.65) | <0.001 | -0.55(-0.79,-0.31) | <0.001 |
| UGMS | 0.04(-0.29,0.38) | 0.79 | -0.14(-0.46,0.19) | 0.41 | -0.12(-0.46, 0.22) | 0.50 |

**Abbreviation:** DI-GM, dietary index for gut microbiota; CAL, clinical attachment loss; BGMS, beneficial to gut microbiota score; UGMS, unfavorable to gut microbiota score; NHANES, National Health and Nutrition Examination Survey; OR, odds ratio; CI, confidence interval; BMI, body mass index.

Model 1 was not adjusted for any covariates.

Model 2 was adjusted for age, sex, and race/ethnicity.

Model 3 was additionally adjusted for BMI, educational attainment, annual household income, smoking status, drinking status, diabetes status, physical activity and total energy intake.

Two-sided *P* values are presented without adjustment for multiple comparisons, with *P* values below 0.001 reported as <0.001.

**Supplementary Table 9.** Associations between DI-GM scores and the proportion of sites with probing pocket depth (PPD) ≥ 4 mm among NHANES 2009–2014 participants.

| Characteristics | Model 1 | | Model 2 | | Model 3 | |
| --- | --- | --- | --- | --- | --- | --- |
|  | *β* (95% CI) | *P* value | *β* (95% CI) | *P* value | *β* (95% CI) | *P* value |
| DI-GM group |  |  |  |  |  |  |
| Q1 | 1(reference) |  | 1(reference) |  | 1(reference) |  |
| Q2 | -0.83(-1.31,-0.35) | <0.001 | -0.62(-1.09,-0.15) | 0.01 | -0.26(-0.72, 0.20) | 0.27 |
| Q3 | -1.23(-1.73,-0.72) | <0.001 | -0.92(-1.42,-0.43) | <0.001 | -0.37(-0.86, 0.12) | 0.14 |
| Q4 | -2.03(-2.50,-1.55) | <0.001 | -1.35(-1.83,-0.88) | <0.001 | -0.51(-0.98,-0.04) | 0.03 |
| P for trend |  | <0.001 |  | <0.001 |  | 0.03 |
| DI-GM score | -0.48(-0.58,-0.37) | <0.001 | -0.33(-0.43,-0.23) | <0.001 | -0.14(-0.24,-0.04) | 0.01 |
| BGMS | -0.58(-0.70,-0.46) | <0.001 | -0.45(-0.57,-0.33) | <0.001 | -0.23(-0.35,-0.10) | <0.001 |
| UGMS | -0.16(-0.33,0.01) | 0.06 | -0.03(-0.20,0.14) | 0.74 | 0.01(-0.17, 0.19) | 0.88 |

**Abbreviation:** DI-GM, dietary index for gut microbiota; PPD: probing pocket depth; BGMS, beneficial to gut microbiota score; UGMS, unfavorable to gut microbiota score; NHANES, National Health and Nutrition Examination Survey; OR, odds ratio; CI, confidence interval; BMI, body mass index.

Model 1 was not adjusted for any covariates.

Model 2 was adjusted for age, sex, and race/ethnicity.

Model 3 was additionally adjusted for BMI, educational attainment, annual household income, smoking status, drinking status, diabetes status, physical activity and total energy intake.

Two-sided *P* values are presented without adjustment for multiple comparisons, with *P* values below 0.001 reported as <0.001.

**Supplementary Table 10. Sensitivity analysis evaluating associations between DI-GM scores (categorized by tertiles) and periodontitis prevalence among NHANES 2009–2014 participants.**

| Characteristics | Model 1 | | Model 2 | | Model 3 | |
| --- | --- | --- | --- | --- | --- | --- |
|  | OR (95% CI) | *P* value | OR (95% CI) | *P* value | OR (95% CI) | *P* value |
| DI-GM group |  |  |  |  |  |  |
| Tertile 1 | 1(reference) |  | 1(reference) |  | 1(reference) |  |
| Tertile 2 | 0.78(0.71,0.85) | <0.001 | 0.77(0.69,0.85) | <0.001 | 0.88(0.79,0.98) | 0.02 |
| Tertile 3 | 0.64(0.57,0.72) | <0.001 | 0.62(0.55,0.70) | <0.001 | 0.81(0.71,0.92) | 0.001 |
| P for trend |  | <0.001 |  | <0.001 |  | <0.001 |

Abbreviation: DI-GM, dietary index for gut microbiota; NHANES, National Health and Nutrition Examination Survey; OR, odds ratio; CI, confidence interval.

Model 1 was not adjusted for any covariates.

Model 2 was adjusted for age, sex, and race/ethnicity.

Model 3 was additionally adjusted for BMI, educational attainment, annual household income, smoking status, drinking status, diabetes status, physical activity and total energy intake.

Two-sided *P* values are presented without adjustment for multiple comparisons, with *P* values below 0.001 reported as <0.001.

**Supplementary Table 11. Additional sensitivity analysis assessing associations between DI-GM scores (categorized by quintiles) and periodontitis prevalence among NHANES 2009–2014 participants.**

| Characteristics | Model 1 | | Model 2 | | Model 3 | |
| --- | --- | --- | --- | --- | --- | --- |
|  | OR (95% CI) | *P* value | OR (95% CI) | *P* value | OR (95% CI) | *P* value |
| DI-GM group |  |  |  |  |  |  |
| Quintile 1 | 1(reference) |  | 1(reference) |  | 1(reference) |  |
| Quintile 2 | 0.89(0.77,1.02) | 0.09 | 0.87(0.75,1.01) | 0.06 | 0.92(0.78,1.07) | 0.28 |
| Quintile 3 | 0.76(0.66,0.87) | <0.001 | 0.76(0.66,0.88) | <0.001 | 0.87(0.75,1.02) | 0.08 |
| Quintile 4 | 0.69(0.60,0.80) | <0.001 | 0.65(0.56,0.76) | <0.001 | 0.79(0.68,0.93) | 0.004 |
| Quintile 5 | 0.6(0.53,0.69) | <0.001 | 0.57(0.50,0.67) | <0.001 | 0.77(0.66,0.90) | <0.001 |
| P for trend |  | <0.001 |  | <0.001 |  | <0.001 |

Abbreviation: DI-GM, dietary index for gut microbiota; NHANES, National Health and Nutrition Examination Survey; OR, odds ratio; CI, confidence interval.

Model 1 was not adjusted for any covariates.

Model 2 was adjusted for age, sex, and race/ethnicity.

Model 3 was additionally adjusted for BMI, educational attainment, annual household income, smoking status, drinking status, diabetes status, physical activity and total energy intake.

Two-sided *P* values are presented without adjustment for multiple comparisons, with *P* values below 0.001 reported as <0.001.

**Supplementary Table 12.** Sensitivity analysis using complete-case data for associations between DI-GM scores and periodontitis prevalence among NHANES 2009–2014 participants.

| Characteristics | Model 1 | | Model 2 | | Model 3 | |
| --- | --- | --- | --- | --- | --- | --- |
|  | OR (95% CI) | *P* value | OR (95% CI) | *P* value | OR (95% CI) | *P* value |
| DI-GM group |  |  |  |  |  |  |
| Q1 | 1(reference) |  | 1(reference) |  | 1(reference) |  |
| Q2 | 0.81(0.72,0.90) | <0.001 | 0.82(0.73,0.93) | 0.001 | 0.93(0.79,1.09) | 0.35 |
| Q3 | 0.74(0.66,0.83) | <0.001 | 0.71(0.62,0.80) | <0.001 | 0.81(0.69,0.95) | 0.01 |
| Q4 | 0.64(0.57,0.72) | <0.001 | 0.62(0.55,0.70) | <0.001 | 0.78(0.67,0.92) | 0.002 |
| P for trend |  | <0.001 |  | <0.001 |  | <0.001 |
| DI-GM score | 0.9(0.88,0.92) | <0.001 | 0.89(0.87,0.92) | <0.001 | 0.94(0.91,0.97) | <0.001 |
| BGMS | 0.84(0.82,0.87) | <0.001 | 0.86(0.83,0.89) | <0.001 | 0.89(0.85,0.93) | <0.001 |
| UGMS | 1.04(1.00,1.08) | 0.04 | 0.98(0.94,1.02) | 0.36 | 1.02(0.96,1.08) | 0.62 |

**Abbreviation:** BMI, body mass index; DI-GM, dietary index for gut microbiota; BGMS, beneficial to gut microbiota score; UGMS, unfavorable to gut microbiota score; NHANES, National Health and Nutrition Examination Survey; OR, odds ratio; CI, confidence interval.

Model 1 was not adjusted for any covariates.

Model 2 was additionally adjusted for age, sex, and race/ethnicity.

Model 3 was additionally adjusted for BMI, educational attainment, annual household income, smoking status, drinking status, diabetes status, physical activity and total energy intake.

Two-sided *P* values are presented without adjustment for multiple comparisons, with *P* values below 0.001 reported as <0.001.

**Supplementary Table 13.** Sensitivity analysis of associations between DI-GM scores and periodontitis prevalence after adjustment for major chronic diseases.

| Characteristics | Model 1 | | Model 2 | | Model 3 | | Model 4 | |
| --- | --- | --- | --- | --- | --- | --- | --- | --- |
|  | OR (95% CI) | *P* value | OR (95% CI) | *P* value | OR (95% CI) | *P* value | OR (95% CI) | *P* value |
| DI-GM group |  |  |  |  |  |  |  |  |
| Q1 | 1(reference) |  | 1(reference) |  | 1(reference) |  | 1(reference) |  |
| Q2 | 0.81(0.72,0.90) | <0.001 | 0.82(0.73,0.93) | 0.001 | 0.92(0.81,1.04) | 0.17 | 0.92(0.81,1.04) | 0.18 |
| Q3 | 0.74(0.66,0.83) | <0.001 | 0.71(0.62,0.80) | <0.001 | 0.83(0.73,0.95) | 0.01 | 0.83(0.73,0.95) | 0.01 |
| Q4 | 0.64(0.57,0.72) | <0.001 | 0.62(0.55,0.70) | <0.001 | 0.81(0.71,0.92) | 0.001 | 0.81(0.71,0.92) | 0.001 |
| P for trend |  | <0.001 |  | <0.001 |  | <0.001 |  | <0.001 |
| DI-GM score | 0.9(0.88,0.92) | <0.001 | 0.89(0.87,0.92) | <0.001 | 0.95(0.92,0.97) | <0.001 | 0.95(0.92,0.97) | <0.001 |
| BGMS | 0.84(0.82,0.87) | <0.001 | 0.86(0.83,0.89) | <0.001 | 0.91(0.88,0.95) | <0.001 | 0.91(0.88,0.95) | <0.001 |
| UGMS | 1.04(1.00,1.08) | 0.04 | 0.98(0.94,1.02) | 0.36 | 1(0.96,1.06) | 0.85 | 1(0.96,1.06) | 0.85 |

**Abbreviation:** BMI, body mass index; DI-GM, dietary index for gut microbiota; BGMS, beneficial to gut microbiota score; UGMS, unfavorable to gut microbiota score; NHANES, National Health and Nutrition Examination Survey; OR, odds ratio; CI, confidence interval.

Model 1 was not adjusted for any covariates.

Model 2 was additionally adjusted for age, sex, and race/ethnicity.

Model 3 was additionally adjusted for BMI, educational attainment, annual household income, smoking status, drinking status, diabetes status, physical activity and total energy intake.

Model 4 was additionally adjusted for the history of hypertension, CVD, and cancer.

Two-sided *P* values are presented without adjustment for multiple comparisons, with *P* values below 0.001 reported as <0.001.

**Supplementary Table 14.** Sensitivity analysis of associations between DI-GM scores and periodontitis prevalence after excluding participants with chronic diseases (N=4,474).

| Characteristics | Model 1 | | Model 2 | | Model 3 | |
| --- | --- | --- | --- | --- | --- | --- |
|  | OR (95% CI) | *P* value | OR (95% CI) | *P* value | OR (95% CI) | *P* value |
| DI-GM group |  |  |  |  |  |  |
| Q1 | 1(reference) |  | 1(reference) |  | 1(reference) |  |
| Q2 | 0.8(0.68,0.95) | 0.01 | 0.78(0.65,0.93) | 0.01 | 0.89(0.74,1.07) | 0.21 |
| Q3 | 0.71(0.60,0.85) | <0.001 | 0.66(0.55,0.80) | <0.001 | 0.82(0.68,1.00) | 0.05 |
| Q4 | 0.6(0.50,0.70) | <0.001 | 0.55(0.46,0.66) | <0.001 | 0.76(0.63,0.92) | 0.01 |
| P for trend |  | <0.001 |  | <0.001 |  | 0.003 |
| DI-GM score | 0.89(0.86,0.92) | <0.001 | 0.87(0.84,0.90) | <0.001 | 0.93(0.90,0.97) | 0.001 |
| BGMS | 0.83(0.80,0.87) | <0.001 | 0.83(0.79,0.87) | <0.001 | 0.89(0.85,0.94) | <0.001 |
| UGMS | 1.02(0.97,1.09) | 0.43 | 0.97(0.91,1.03) | 0.35 | 1.01(0.94,1.09) | 0.74 |

**Abbreviation:** BMI, body mass index; DI-GM, dietary index for gut microbiota; BGMS, beneficial to gut microbiota score; UGMS, unfavorable to gut microbiota score; NHANES, National Health and Nutrition Examination Survey; OR, odds ratio; CI, confidence interval.

Model 1 was not adjusted for any covariates.

Model 2 was additionally adjusted for age, sex, and race/ethnicity.

Model 3 was additionally adjusted for BMI, educational attainment, annual household income, smoking status, drinking status, physical activity and total energy intake.

Two-sided *P* values are presented without adjustment for multiple comparisons, with *P* values below 0.001 reported as <0.001.
